# Precision Family Spirit: a pilot randomized implementation trial of a precision home visiting approach with families in Michigan—trial rationale and study protocol

**DOI:** 10.1186/s40814-020-00753-4

**Published:** 2021-01-06

**Authors:** Allison Ingalls, Allison Barlow, Elizabeth Kushman, Amanda Leonard, Lisa Martin, Precision Family Spirit Study Team, Allison L. West, Nicole Neault, Emily E. Haroz

**Affiliations:** 1 Center for American Indian Health, Department of International Health, Johns Hopkins Bloomberg School of Public Health, Baltimore, MD USA; 2 Maternal, Infant and Early Childhood Services, Inter-Tribal Council of Michigan, Inc., Sault Sainte Marie, MI USA; 3 Department of Population, Family and Reproductive Health, Johns Hopkins Bloomberg School of Public Health, Baltimore, MD USA

**Keywords:** Precision science, Precision public health, Implementation science, Hybrid design, American Indian, Pregnancy and childbirth, Randomized controlled trials, Home-visiting

## Abstract

**Background:**

Home visiting is a well-supported strategy for addressing maternal and child health disparities. However, evidence-based models generally share implementation challenges at scale, including engagement and retention of families. Precision home visiting may address this issue. This paper describes the first known pilot randomized implementation trial of a precision home visiting approach vs. standard implementation. Primary aims are to: 1) explore the acceptability and feasibility of a precision approach to home visiting and 2) examine the difference between *Standard Family Spirit* and *Precision Family Spirit* on participants’ program satisfaction, client-home visitor relationship, goal alliance, and the impact of these factors on participant engagement and retention. Secondary aims are to explore potential differences on maternal behavioral and mental health outcomes and child development outcomes to inform sample size estimations for a fully powered study.

**Methods:**

This is a pilot Hybrid Type 3 implementation trial. Four Michigan communities primarily serving the Native American families and already using *Family Spirit* were randomized by site to receive Standard Family Spirit or Precision Family Spirit. Participants include *N* = 60 mothers at least 14 years of age (pregnant or with a newborn < 2 months of age) currently enrolled in Family Spirit. Precision Family Spirit participants receive core lessons plus additional lessons based on needs identified at baseline and that emerge during the trial. Control mothers receive the standard sequence of Family Spirit lessons. Data is collected at baseline (< 2 months postpartum), and 2, 6, and 12 months postpartum. All Precision Family Spirit participants are invited to complete qualitative interviews at study midpoint and endpoint. All home visitors are invited to participate in focus groups between study midpoint and endpoint. Exploratory data analysis will assess feasibility, acceptability, client-home visitor relationship, retention, adherence, and potential differences in intervention outcomes.

**Discussion:**

This trial will provide new information about the acceptability and feasibility of precision home visiting and pilot data on program satisfaction, client-home visitor relationship, goal alliance, retention, and targeted maternal-child intervention outcomes. Findings will inform the design of a fully powered randomized implementation trial of precision vs. standard home visiting.

**Trial registration:**

ClinicalTrials.gov #NCT03975530; Registered on June 5, 2019

**Supplementary Information:**

The online version contains supplementary material available at 10.1186/s40814-020-00753-4.

## Key messages on feasibility

- Prior to this pilot study, it is unclear whether comparing a modularized version of a home visiting program to standard practice home visiting would be feasible, and, if so, whether there would be detectable differences between the approaches.
- Results will inform whether and how to design a fully powered randomized controlled trial to test implementation effectiveness of a modularized version of home visiting to improve primary outcomes*.*
- The results of the study will inform 1) how to approach randomization, 2) data collection platforms to use, 3) sample size calculations, and 4) adaptations to the design and measures for a fully powered randomized controlled trial.

## Background

Decades of research validate early childhood home visiting as an effective strategy to support families. Home visiting programs target a range of maternal and child outcomes, including promoting healthy growth and development, and reducing maternal and child behavioral and mental health disparities. In 2010, the United States Congress authorized $1.5 billion to fund the Maternal, Infant, and Early Childhood Home Visiting (MIECHV) Program across the United States (US). MIECHV was reauthorized and funded at $400 million per year for 5 years in early 2018. The Home Visiting Evidence of Effectiveness (HomVEE) review identified 21 home visiting models that meet the US Health and Human Services criteria for an evidence-based early childhood home visiting service delivery model. Of these, 19 are eligible to be used with MIECHV funds [1, 2].

Evidence-based home visiting programs being disseminated across the US struggle with retention of families. A recent report indicated only half of participating families across four home visiting models remained in the program at one year postpartum, despite these models expecting that families remain enrolled until at least 2 years postpartum [3]. Many home visiting models were designed to serve families over several years with regularly scheduled visits. While some shorter home visiting models have seen positive outcomes, little is known regarding optimal duration and dosage [4]. Moreover, the types of families being served and the diversity of issues and challenges they present suggest the need to tailor and adapt programs to better fit a wide range of needs [5]. To date, this tailoring has almost always relied solely on the judgement of an individual home visitor or, in some cases, is guided by the implementing organization without guidance from the model developer.

Recent qualitative research on high- and low-retention sites using one evidenced-based home visiting model showed that nurses in high-retention sites were more collaborative with families. They were more likely to adapt their program delivery to align with families’ needs, compared to nurses in low-retention sites [6]. Based on these findings, Ingoldsby and colleagues (2013) developed an implementation strategy grounded in the principals of motivational interviewing to provide nurses with more explicit control over the visit schedule and content to families. When they compared retention rates for nurses trained in motivational interviewing to rates for nurses trained in the standard approach, preliminary findings showed retention rates were significantly better when nurses had more flexibility in service delivery [7]. It is important to note that authors recognized several other factors that may be influencing these positive findings. Nonetheless, improving implementation of programs at scale including achieving high participant retention, enhancing program content to address families’ unique and complex needs, and increasing model fidelity is of key interest to researchers, implementing agencies, and other stakeholders [8].

From 2016 through 2018, the Johns Hopkins Center for American Indian Health (CAIH) conducted formative work to develop an implementation strategy for *Family Spirit®* based on concepts of design modularity [9]. Design modularity aims to systematically tailor content to meet families’ needs. Family Spirit is a federally endorsed, evidence-based model designed for, by, and with Native American communities [10–12]. The rationale for the development of a precision implementation strategy for Family Spirit, called Precision Family Spirit, was based on two factors. First, home visitors are faced with the complex task of applying knowledge gained from previous research studies to a diverse range of clients who may be substantially different from the populations from which this research was generated. This yields potential problems with fidelity. Second, based on studies indicating tailoring may help with retention [6, 7], we sought to use modularization as an implementation strategy to tailor Family Spirit while maintaining fidelity to the model [13].

The current study is a pilot Hybrid Type 3 [14] implementation trial of Precision Family Spirit. This hybrid design tests an implementation strategy while also gathering data on relevant outcomes. This pilot study will examine the acceptability, feasibility, and preliminary effects of a “precision approach” to home visiting. Our specific aims are to 1) explore the acceptability and feasibility of a precision approach to home visiting from the perspective of enrolled mothers and 2) examine the difference between *Precision Family Spirit* and *Standard Family Spirit* on program satisfaction, client-home visitor relationship, goal alliance, and retention. As part of the hybrid design, we will also monitor client level intervention effects on maternal self-efficacy, stress, and child social, emotional, and behavioral development.

## Methods/design

### Trial design

**Fig. 1:**
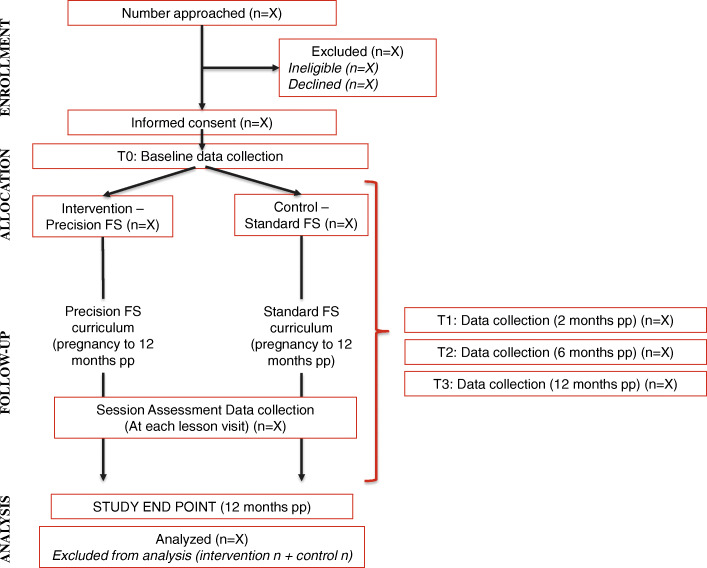
Study design and data collection

This study uses a two-arm pilot randomized Hybrid Type 3 design [14]. Four Family Spirit home visiting sites from the Inter-Tribal Council of Michigan (ITC of MI) were selected based on comparability and randomized using a 1:1 allocation to provide either Standard Family Spirit or Precision Family Spirit to their clients. Sites were matched based on annual volume of clients served and geographic similarity (i.e., urban vs. rural) (Fig. 1).

### Participants

Our target population for recruitment is women who are *either* pregnant or have a baby no older than 2 months of age. All study participants must be at least 14 years of age and already enrolled in the Family Spirit program in their community. Since 2013, the CAIH and the ITC of MI have partnered on implementing the Family Spirit model across participating tribal communities. The ITC of MI has a strong infrastructure to support the incorporation of a research study into their existing home visiting programming.

### Participating communities

Four ITC of MI sites are participating in this pilot trial, all located in the US and primarily serving Native American families—two in the Upper Peninsula of Michigan (MI) and two in the Lower Peninsula of MI, one of which is an Urban Indian Organization (UIO). The ITC of MI was founded in 1968 to combine efforts of a few Native American tribal communities. Working collaboratively allowed these tribal communities to leverage resources and expand programs to enhance the health and well-being of tribal members. Today, the ITC of MI is a consortium of all 12 federally recognized tribes and 1 UIO in Michigan. Member tribes directly manage local program operations while ITC of MI provides technical assistance and administrative support, often serving as grantee on projects involving a consortium of tribes. To respect tribal sovereignty and their wishes, the participating Tribes’ names have been left out of this paper.

Native American communities in the Upper Peninsula are rural and geographically isolated. While a sizeable portion of the population lives on reservation land in relative proximity to tribal services, many live off reservation across a wide area of multiple counties. Historical trauma and government policies which disrupted tribal communities’ economic, social, and cultural structures and overall way of life have led to disparities across income, employment, health, and wellness compared to the Michigan general population. Likewise, Lower Peninsula tribal communities face socioeconomic and health disparities as a result of historical trauma and government policies. While Lower Peninsula communities are characterized as more semi-urban and urban as compared to those in the Upper Peninsula, their families are facing similar challenges. Thus, both Lower and Upper Peninsula communities have been using home visiting as a part of a larger strategy to strengthen families.

### Family Spirit Model

The Family Spirit evidence-based home visiting model was designed and tested by the CAIH in partnership with tribal communities over a 20-year period [10]. It is currently the largest, most rigorous, and only evidence-based home visiting program designed for pregnant and parenting Native American families. Family Spirit trains Native American community members to serve as home visitors who provide structured, culturally grounded education to families with children ages 0 to 3. The goal is to produce healthier pregnancies and healthier future generations by providing education around pregnancy, parenting, child development, and life skills and connecting families with community resources. The model was designed for delivery by paraprofessionals but can also be delivered by professionals. The employment of paraprofessionals to deliver the program is more sustainable and builds capacity within communities that suffer low employment and poor access to health care and education. It also allows for cultural match between home visitors and participants, as most professionals in the communities are non-Native.

Family Spirit utilizes a 63-lesson curriculum, which was designed through a community-based participatory process led by the CAIH in partnership with three Southwestern Native American tribal communities. The original curriculum was evaluated through three successive randomized controlled trials (RCTs), each one increasing in length and rigor and corroborating prior findings. In the third and largest RCT, all families in the intervention study arm received all Family Spirit lessons. Lesson visits typically lasted an hour and started out weekly and decreased in frequency over time, until they were being taught bimonthly from 2 to 3 years postpartum. Family Spirit has demonstrated the following impacts: increases in parenting knowledge [10–12, 15] and self-efficacy [10, 15]; reductions in parenting stress [12, 15] and maternal psychosocial risks that could impede positive parenting, including substance use [15] and depression [11, 12, 15]; and improved children’s social, emotional, and behavioral development [10, 12, 15]. Based on these findings, in 2013, Family Spirit received the highest ratings for an “evidence-based” program from the federal HomVEE review of home visiting program effectiveness and a 4.0 out of 4.0 for dissemination readiness from the National Registry for Evidence-Based Practices and Programs (NREPP). At present, over 130 tribal communities and 4 urban non-Native communities across 22 US states have been trained to deliver Family Spirit*.* The program has been adopted by non-Native communities due to its unique cultural strengths, use of paraprofessionals, and strong evidence of effectiveness. Family Spirit respects tribal data sovereignty and thus does not report nationally on retention of participating families. However, like other home visiting models being disseminated across the US, Family Spirit affiliates have shared challenges with retention and engagement of families for the duration of the program. A precision approach to Family Spirit provides promise to addressing this issue of implementation of an evidence-based home visiting program at scale.

### Recruitment

The pilot study is recruiting *N* = 60 women who meet the inclusion and exclusion criteria below:

### Inclusion criteria

1. Women who are *either* pregnant or have a baby no older than 2 months old
2. At least 14 years of age at time of conception
3. Enrolled in Family Spirit services in the participating community

### Exclusion criteria

1. Inability to participate in full intervention or evaluation (e.g., planned move, scheduling conflicts, etc.)

Ambiguous cases based on unclear age at conception or unclear gestational age are reviewed by the Study Management Team before being deemed eligible for recruitment. Home visitors conduct informed consent procedures with all eligible participants. All participants must provide informed consent or assent to participate. Participants under age 18 must have parent/guardian consent. The first participant gave consent on June 25, 2019, and the first baseline assessment was completed on June 26, 2019.

### Randomization

Participating ITC of MI sites were randomized to use the Precision Family Spirit implementation strategy or Standard Family Spirit (control condition). Randomization occurred before recruitment began at the site level. The four sites were selected based on comparability of annual volume of clients served and geographic similarity (i.e., urban vs. rural). Once sites were matched, the study team flipped a coin to decide which sites would be assigned to each study arm. Participants are blind to whether they receive Standard Family Spirit or Precision Family Spirit as randomization occurs at the site-level. Thus, all participants at each site receive the same version of Family Spirit (standard or precision).

### Ethical considerations

This study was reviewed and approved by the Johns Hopkins Bloomberg School of Public Health Institutional Review Board (JHBSPH IRB) (IRB00009315; Current protocol version: #9, approved on September 8, 2020). Any protocol modifications are submitted as an amendment to the JHBSPH IRB. Supervisors then inform study staff and conduct additional trainings as needed. If protocol modifications are significant, trial registries are updated.

Serious adverse events (e.g., participant death or hospitalization) for both mothers and/or children are reported on a real-time basis to the JHBSPH IRB. All study investigators have been trained and certified in basic human subject research, HIPAA compliance, and Social and Behavioral Research Best Practices for Clinical Research. All home visitors have also been trained and certified in HIPAA compliance.

### Standard Family Spirit

Mothers at the control sites receive Family Spirit as usual, per existing ITC of MI program policies and procedures. In the standard of care, families receive regular home visits, delivered in the same sequence until 3 years postpartum (63 lessons total). Because the duration of enrollment for this pilot study is from pregnancy to 12 months postpartum, Standard Family Spirit participants should receive 47 lessons total. Home visitors at Standard Family Spirit sites use the Care4 platform to track their clients. They receive notifications about high depression scores, risk of self-harm, and potential domestic violence issues, but they do not receive notifications about other risk categories (first-time mom, substance use, nutrition, sexual and reproductive health) that would inform tailoring sequences in the Precision Family Spirit implementation approach. Details on the rationale for selecting these risk categories have been described elsewhere [9, 16].

In both arms of the study, home visitors provide referrals and connections to community agencies and services as needed. Existing procedures are in place to link participants to assistance that is available in their area. Home visitors often act as navigators and advocates for the families they serve. In addition, participant transportation to and from doctor and clinic appointments is common and will continue for enrolled participants in each arm.

The study team recognizes that differences may be small regarding number of prescribed lessons between Precision Family Spirit and Standard Family Spirit participants, but implementation outcomes may be potentially detectable because we are measuring program participant acceptability, satisfaction, quality of relationship between home visitor and client, retention, and adherence.

### Implementation strategy: Precision Family Spirit

Our selection of an implementation strategy is guided by the Proctor et al. 2009 conceptual model of implementation [17]. Home visiting models in practice struggle with low retention rates [3]. Thus, we used design modularity as an implementation strategy that would improve engagement and retention. The specification of our implementation strategy can be found in Table 1 [18].

**Table 1:** Specification of Family Spirit implementation strategy

Domain	Strategy: modularization of an EBT
Actor(s)	Program development team; Implementers
Action(s)	Generate appropriate lesson pathways; implement these using an electronic support platform (Care4)
Target(s) of the action	Home visitors implementing Family Spirit; Clients receiving Family Spirit
Temporality	During the entire delivery of the program
Dose	Continuous
Implementation outcomes affected	Penetration (engagement and retention) among eligible clients; Adherence to the active prescribed ingredients of Family Spirit
Justification	Research suggests that retention is low in home visiting programs and tailoring of the program improves engagement and retention.

*Note:* Guidelines for specification of the *Family Spirit* implementation strategy from Proctor et al. (2013)

This modularized approach to Family Spirit is called Precision Family Spirit. Precision Family Spirit was developed using mixed methods, including a secondary data analysis to identify meaningful subgroups that could inform how and when to tailor, and stakeholder workshops with program implementers (supervisors and home visitors) to identify core lessons, and lessons relevant to certain subgroups of clients [9, 16]. It consists of five lesson “pathways” that have been identified through formative work across Family Spirit’s national network of stakeholders [9]. There is a core set of lessons that every mother receives. For this pilot study, that means all Precision Family Spirit participants should receive 25 lessons from pregnancy to 12 months postpartum (the duration of enrollment). She receives additional lessons based on her emergent needs identified through data collection at home visits and at each assessment timepoint (Additional file 1). Thus, a Precision Family Spirit participant could be scheduled to receive between 25 and 41 lessons from pregnancy to 12 months postpartum. For example, if a participant is a first-time mother, she (and her family) will receive 6 additional baby care lessons (covering basic topics such as diapering and how to dress a baby) that are included in the Standard Family Spirit curriculum but may not be relevant for experienced mothers with previous children.

The study uses the Care4 implementation platform, which is a secure, HIPAA-compliant web-based application designed to capture data for the purposes of program implementation and evaluation [19]. Care4 is configured to notify home visitors when a participant needs to be added to any additional lesson pathways. Precision Family Spirit participants are assessed at each lesson visit using a measurement-based care approach [20–22] to understand and respond to emergent needs and help home visitors better track how their clients are doing over time. These short assessments are estimated to take 1–2 min to complete at each visit. Lessons are delivered in the same format as Standard Family Spirit, by trained home visitors in the home of the participant or a private place of their choosing.

### Data collection time points

Participant assessments include maternal self-reports using Care4 and an interview (Table 2). Self-reports are conducted in participants’ homes, another private location, or via text/email on an as needed basis. Data is recorded on either a tablet, computer, or paper. If data is collected on paper, the home visitor enters the data into Care4 upon her return to the office. In some cases, assessments are conducted via text or email, once the home visitor discusses on a case by case basis with the study management team. For example, if a home visit is cut short, assessments may be completed post-visit by the participant. A secure link is sent via text or email with instructions on how to complete and submit. All participants will be assessed at four timepoints: baseline, 2 months, 6 months, and 12 months postpartum. Each assessment timepoint is estimated to take participants between 30 min and 1 h to complete. In an effort to mitigate participant time burden, home visitors complete assessments with participants during already-scheduled visits, by scheduling a separate visit, or by emailing or sending links to participants via SMS to complete the forms on their own time. Precision Family Spirit participants may also be asked to participate in a phone interview at study midpoint (between 4.5 and 6.5 months postpartum) and study endpoint (between 11 and 13 months postpartum), adding an additional 15–20 min of participant time burden for each interview completed. The purpose of these interviews is to further contextualize data being collected as part of the quantitative assessment.

**Table 2:** List of hybrid trial objectives and evaluation measures

Pilot trial objectives	Instrument	Timepoint			
		Baseline	2 months postpartum	6 months postpartum	12 months postpartum
**Baseline characteristics**					
N/A	Maternal demographics	X			
**Primary objectives**					
Acceptability	Program acceptability			X	X
	Qualitative interview**			X	X
Satisfaction	Participant satisfaction			X	X
	Qualitative interview**			X	X
Home visitor-participant relationship	Working alliance inventory: 1. Client WAI 2. Home visitor WAI		X	X	X
Retention	Participant tracking information collected throughout the duration of the pilot study				
Adherence					
**Secondary objectives**					
Maternal substance use	Alcohol, Smoking and Substance Involvement Screening Test	X	X	X	X
Maternal depression	Edinburgh Postnatal Depression Scale	X	X	X	X
Parenting feeding practices	Child Feeding Assessment		X	X	X
Maternal parental knowledge	Parent Knowledge Assessment	X	X	X	X
Child development	Ages and Stages: Social Emotional - 2*		X	X	X
	Ages and Stages Questionnaire, 3rd Edition*		X	X	X
Quality of life	EURO-QOL	X	X	X	X
Maternal self-efficacy	Parenting Locus of Control			X	X
Maternal stress	Perceived Stress Scale 4	X	X	X	X
Substance abuse/domestic violence risk	Institute for Health and Recovery Integrated Screening Tool	X	X	X	X
Client priority outcomes	Top problems	X	X	X	X

*These measures will not be administered as part of the Care4 data collection platform, as they are licensed by ITC of MI external to the Care4 platform

**Qualitative interviews will only be conducted with intervention participants at midpoint and endpoint

Participants receive a gift card upon completion of the following assessment timepoints: baseline, 2 months, 6 months, and 12 months postpartum. Total possible remuneration for participants in the study is $70 in gift cards.

From study midpoint until the endpoint of the study timeline, home visitors will be asked to participate in focus group discussions (FGDs) by video conference. Each site will participate in up to 10 short FGDs. FGDs will be designed to take approximately 30 min to complete and will be scheduled during regular study site meetings. They will be recorded with permission and transcribed verbatim. Home visitors receive a $15 gift card upon completion of each FGD. Total possible remuneration for home visitors is $150 in gift cards.

### Outcomes

Selected measures are being used to assess implementation and clinical outcomes (Table 2).

#### Primary outcomes

Our primary outcomes of interest include participant acceptability, satisfaction, quality of relationship between home visitor and client, retention, and adherence. Acceptability is measured from the perspective of the participant using a 15-item scale [16] administered at study midpoint and endpoint. Satisfaction is captured through a 34-item questionnaire used in the Family Spirit trials and adapted specifically for this study and administered at midpoint (around 6 months postpartum) and endpoint. All Precision Family Spirit participants are invited to participate in a short qualitative interview to further explore acceptability and satisfaction using an 11-item interview guide. In addition, we will explore home visitor acceptability and satisfaction through focus group discussions that follow a 9-item interview guide. The quality of the relationship between home visitor and client is captured using the short version of the Working Alliance Inventory (WAI) administered to both participant and home visitor. The WAI measures three domains of the relationship: goal alignment, task alignment, and bond. Retention is measured by comparing the number of participants who drop out in each study arm. Adherence is measured by comparing the percentage of retained participants in each study arm who completed their lessons as prescribed.

#### Secondary outcomes

As part of the Hybrid Type 3 approach, we also monitor specific maternal behavioral and mental health outcomes and child physical and social-emotional development outcomes.

Maternal substance use is measured at all assessment timepoints (baseline, 2, 6, and 12 months postpartum) using an adapted version of the World Health Organization’s validated Alcohol, Smoking and Substance Involvement Screening Test (ASSIST) [23, 24]. The ASSIST screens for all levels of problem or risky substance use (alcohol, illegal drugs, prescription drugs, and tobacco use). A risk score is provided for each substance, and scores are grouped into low, moderate, or high risk.

Maternal depression is measured at all timepoints (baseline, 2, 6, and 12 months postpartum) using the validated Edinburgh Postnatal Depression Scale (EPDS) [25]. The EPDS is a 10-item self-rating scale that was specifically designed for women who are pregnant or have just had a baby. However, it has also been shown to be an effective measure for general depression in the larger population [26].

Parent feeding practices are measured at 2, 6, and 12 months postpartum using an assessment developed by the study team. It is adapted from a child feeding assessment used in two other CAIH randomized trials in Southwest Native American communities [27, 28]. The assessment will be used to assess feeding practices, introduction of complementary feeding and introduction of sugar sweetened beverages among newborns and infants.

Maternal parenting knowledge is measured at all timepoints (baseline, 2, 6, and 12 months postpartum) using a 10-item assessment developed by the study team and tied to core Family Spirit curriculum content.

Other maternal outcomes are measured at all timepoints (baseline, 2, 6, and 12 months postpartum) using short screening tools or measures: overall quality of life is measured using the EuroQol [29], maternal self-efficacy is measured using the Parental Locus of Control scale [30], parental stress is measured using the Perceived Stress Scale 4 [31], substance abuse/domestic violence risk is measured using the Institute for Health and Recovery Integrated Screening Tool [32], and client priority outcomes are measured using the Top Problems measure [33]. Each measure included in this assessment is valid and reliable, with some having been shortened through a previous secondary data analysis using Item Response Theory Methods [34].

Child development is measured using the parent-report, validated Ages & Stages Questionnaire (ASQ-3) [35] and Ages & Stages Questionnaire: Social-Emotional, Second Edition (ASQ:SE-2) [36]. The ASQ-3 is used to monitor all five domains of child development and screen children for developmental delays during the first 5 years of life. The ASQ:SE-2 focuses on social-emotional development of the child. The ITC of MI uses the ASQ-3 and ASQ:SE-2 as part of ongoing programmatic data collection. The CAIH will export sum scores at 2, 6, and 12 months postpartum for both assessments to use in data analysis.

#### Quality assurance

To ensure quality of participant consent procedures, all study staff have been certified to consent participants. Home visitors received initial in-person training on informed consent. Post-training, they had to demonstrate mastery of a full informed consent procedure with their supervisor prior to being certified. Program supervisors used a checklist to certify home visitors at their site.

Quality assurance of lesson delivery for both study arms occurs according to existing ITC of MI procedures. All home visitors are required to pass knowledge tests on each lesson prior to administering them to clients. Further, they must complete role plays and achieve a total average score of at least 3 out of 4 on a quality assurance form administered by a supervisor or program manager. To ensure ongoing high quality of lesson delivery, home visit observations are conducted at least biannually and ideally quarterly. For home visitors with at least 5 years of experience implementing Family Spirit, they are observed annually or more frequently if desired by the home visitor. Home visitors participating in this pilot trial are, in general, experienced in delivering Standard Family Spirit*.* Of 13 trained home visitors, 8 (62%) have at least 5 years of experience as Family Spirit certified home visitors. Further, 11 (85%) have at least 3 years of experience. All home visitors have been trained for at least a year before this pilot trial launched.

#### Quality control of data management

Assessment forms are initially checked for completion by the home visitor. All data are directly entered into the Care4 platform and stored in their secure electronic database. Weekly quality control procedures are carried out by the research coordinator to identify specific errors and missing data. The study statistician carries out quality control routines on an as needed basis in R. All R data sets are stored on a secure server. The research coordinator communicates all data queries to home visiting staff via email or on biweekly site calls.

#### Sample size calculation and statistical analysis

As this is a pilot study, our target sample (*N =* 60) was selected on the basis of feasibility of study completion, representativeness of the target study population (includes urban and rural sites) and with the desire to provide useful information to inform a fully powered research study (e.g., inclusion/exclusion criteria, variability in responses, estimated effects). Even so, with significance set to *a =* 0.05 and power set to 80%, using our *N =* 60 and accounting for 30% attrition, we would expect to be able to detect a large effect (*d =* 0.8) on continuous outcomes and a 35% difference in retention rates between the two groups.

#### Primary aim analysis

As this is a pilot study, most data analysis will be exploratory, but based on best practices in the field to analyze clinical trial data. We will conduct an “intent to treat” analysis as the primary analysis approach. Baseline demographic characteristics will be explored to examine comparability between the groups. For continuous measures, we will explore average scores and distributions at each time point. To test for effects, we will use mixed-effects regression models (MRMs) to estimate average differences between outcomes on the standard vs. precision approaches using the scores on the acceptability, participant satisfaction, and home visitor-client relationship assessments. Specifically, we will add a dichotomous indicator for participants receiving the precision approach compared to the standard approach and examine the interaction of that dichotomous indicator with the change in outcome measure. The MRMs will include random effects at the individual and site levels to account for multiple time points within an individual and clustering by site. We will also examine the differences in proportion of participants who dropped out and who received 50% dosage using mixed effects logistic regression models accounting for clustering by site. For all analyses, we will report both unadjusted and adjusted analyses (adjustment based on differences in baseline demographics between the two groups at the *p <* 0.1 level). For all qualitative data, we will use thematic analysis to analyze interview transcripts using Dedoose software [37]. As transcripts are reviewed, “memos” will be generated to document initial impressions of topics and themes. Segments of transcripts will then be assigned codes based on a priori (i.e., from the interview guide) or emergent themes (i.e., open coding) [38].

All analyses for the secondary outcomes will be done on an exploratory basis only.

## Discussion

This is the first pilot randomized implementation trial of precision home visiting. We used the principals of design modularity to develop an implementation strategy to help guide home visitors in what to deliver and when, with the goal of improving retention and engagement while maintaining fidelity to what we know works. Our implementation strategy was developed through participatory methods with program implementers. This pilot implementation study will provide important preliminary understanding into the acceptability and feasibility of applying this precision approach to evidence-based home visiting, a major priority for the national home visiting field [5]. All evidence-based home visiting models incorporate content delivery into their implementation strategies, which is what this study focused on [13]. As such, authors see promise in translating findings and lessons learned from this pilot study into other models wishing to design precision approaches to home visiting.

### Limitations

There are some limitations to this pilot study. First, we focused on content delivery (i.e., prescribed active ingredient) of the Family Spirit model. However, the home visitor-client relationship (e.g., unprescribed active ingredient) could be the focus of implementation strategies in home visiting [7, 39]. Results of data collected in this pilot trial through the Working Alliance Inventory will help us explore the quality of the home visitor-client alliance to understand whether tailoring content impacts this relationship.

Second, there was a small number of groups randomized into each study arm. Only four ITC of MI sites were included in this pilot trial. Therefore, we are unlikely to achieve completely comparable groups. However, within the context of these limitations, the results of this pilot trial can inform a fully powered cluster randomized trial in the future.

Third, there are some concerns regarding generalizability of study findings. Yet, a strength of this pilot study is that was designed for Native American communities, which are the most historically disenfranchised racial/ethnic subgroup in the US facing substantial behavioral health and sociodemographic disparities [40–42]. Positive findings in this population may provide promise for translation into other communities.

## Conclusion

This pilot Hybrid Type 3 implementation study will provide a better understanding of how precision home visiting can be used to increase participant engagement and retention and provide insights as to whether outcomes can be strengthened by a precision approach. The results from this study can help refine the implementation and evaluation methodologies and inform a fully powered hybrid trial to test the effectiveness of a precision implementation strategy on improved participant retention and outcomes. Ultimately, this line of research will advance our understanding of how most efficiently and effectively to support the healthy growth and development of families with a wide range of needs.

## Supplementary Information

**Additional file 1:.** Precision Family Spirit Lesson Pathways. Title of data: Precision Family Spirit Lesson Pathways – Pregnancy to 12 Months Postpartum. Description of data: Details of the five Precision Family Spirit pathways (pregnancy-12 months postpartum), including period and timepoint

**Additional file 2:.** SPIRIT-CONSORT Extension to Pilot Trials. Title of data: Adapted and combined SPIRIT 2013 Checklist and CONSORT Extension to Pilot Trials. Description of data: An adaptation of recommended items to address in a clinical trial protocol and related documents

## Data Availability

Not applicable

## Abbreviations

AIHFS: American Indian Health and Family Services
ASQ-3: Ages and Stages Questionnaires
ASQ:SE-2: Ages & Stages Questionnaire: Social-Emotional, Second Edition
ASSIST: Alcohol, Smoking and Substance Involvement Screening Test
CAIH: Johns Hopkins Center for American Indian Health
EPDS: Edinburgh Postnatal Depression Scale
FGD: Focus group discussion
HomVEE: Home Visiting Evidence of Effectiveness
ITC of MI: Inter-Tribal Council of Michigan
JHBSPH IRB: Johns Hopkins Bloomberg School of Public Health Institutional Review Board
MIECHV: Maternal, Infant, and Early Childhood Home Visiting Program
MRM: Mixed-effects regression model
RCT: Randomized controlled trial
UIO: Urban Indian Organization
US: United States
WAI: Working Alliance Inventory
