# Supplementary material for: Precision Family Spirit: a pilot randomized implementation trial of a precision home visiting approach with families in Michigan—trial rationale and study protocol
Source: Pilot Feasibility Stud. 2021 Jan 6;7:8. doi: 10.1186/s40814-020-00753-4 (PMC7786970; doi:10.1186/s40814-020-00753-4)
Supplement: Supplementary file 1 — Additional file 1:. Precision Family Spirit Lesson Pathways. Title of data: Precision Family Spirit Lesson Pathways – Pregnancy to 12 Months Postpartum. Description of data: Details of the five Precision Family Spirit pathways (pregnancy-12 months postpartum), including period and timepoint [file 40814_2020_753_MOESM1_ESM.docx]

Additional File 1. Precision Family Spirit Lesson Pathways – Pregnancy to 12 Months Postpartum

| Timepoint | Lesson Pathways | | | | |
| --- | --- | --- | --- | --- | --- |
|  | Core Lessons (n=25) | First Time Mom (n=6) | Substance Abuse Risk (n=2) | Childhood Obesity Risk (n=5) | Sexual/ Reproductive Health (n=3) |
| 28 weeks gestation | Contributing to a Healthy Pregnancy |  |  |  |  |
| 29 weeks gestation |  |  | Effects of Drug Use on a Developing Baby |  |  |
| 30 weeks gestation | Working Towards a Better You |  |  |  |  |
| 31 weeks gestation |  |  |  | What You Eat = Your Baby’s Future |  |
| 32 weeks gestation | Bedtime Safety |  |  |  |  |
| 33 weeks gestation |  |  |  |  |  |
| 34 weeks gestation | How to Feed |  |  |  |  |
| 35 weeks gestation |  | How to Diaper; How to Dress; How to Bathe |  |  |  |
| 36 weeks gestation | Breastfeeding Basics |  |  |  |  |
| 37 weeks gestation |  | Preparing for Safe Travel; Before and During Labor |  |  |  |
| 38 weeks gestation | After Your Baby is Born |  |  |  |  |
| 1 week postpartum |  |  |  | Feeding Support |  |
| 2 weeks postpartum | How to Comfort Your Crying Child |  |  |  |  |
| 3 weeks postpartum |  | What to Do if Your Baby is Sick |  |  |  |
| 4 weeks postpartum | Parenting Techniques Part A* |  |  |  |  |
| 5 weeks postpartum |  |  |  |  | Understanding Reproduction |
| 6 weeks postpartum | How to Protect |  |  |  |  |
| 7 weeks postpartum |  |  |  |  | Your Family Planning Options |
| 8 weeks postpartum | Planning Ahead |  |  |  |  |
| 9 weeks postpartum |  |  |  | Rethink that Drink |  |
| 10 weeks postpartum | Playtime Fun; Protecting Children from Abuse and Neglect |  |  |  |  |
| 12 weeks postpartum | Parenting Techniques Part B* |  |  |  |  |
| 14 weeks postpartum | Introduction to Oral Health Care |  |  |  |  |
| 16 weeks postpartum | Introducing Solid Foods to Your Baby |  |  |  |  |
| 18 weeks postpartum | Communication and Building Healthy Relationships |  |  |  |  |
| 20 weeks postpartum | Skills for Healthy Living Part A |  |  |  |  |
| 22 weeks postpartum |  |  |  | Infant Physical Activity and Safe Play Space |  |
| 24 weeks postpartum | Skills for Healthy Living Part B |  |  |  |  |
| 7 months postpartum | Your Baby’s Developing Senses |  |  |  |  |
| 7.5 months postpartum | A Look at Drug Use in Our Community |  |  |  |  |
| 8 months postpartum | Beginning Conversations |  |  |  |  |
| 8.5 months postpartum | Baby Proofing and Safety |  |  |  |  |
| 9 months postpartum | Becoming More Mobile |  |  |  |  |
| 9.5 months postpartum |  |  |  | Finger Foods for Babies |  |
| 10 months postpartum | Understanding Cause and Effect |  |  |  |  |
| 10.5 months postpartum |  |  | Effects of Drug Use on Our Families |  |  |
| 11 months postpartum | Developing Hand-Eye Coordination |  |  |  |  |
| 11.5 months postpartum |  |  |  |  | Protecting Your Sexual Health |
| 12 months postpartum | Development of Memory Skills |  |  |  |  |
| *Parenting Techniques Parts A & B are considered one lesson in the *Family Spirit* curriculum. | | | | | |
